# Use of pudendal nerve block among midwives in Norway: A national cross-sectional study

**DOI:** 10.18332/ejm/146690

**Published:** 2022-06-07

**Authors:** Mirjam Lukasse, Alette B. Bratsberg, Katrine Thomassen, Ellen A. Nøhr

**Affiliations:** 1 Department of Nursing and Health Sciences, Faculty of Health and Social Sciences, University of South-Eastern Norway, Kongsberg, Norway; 2 Department of Obstetrics, Telemark Hospital Trust, Skien, Norway; 3 Department of Obstetrics, Vestfold Hospital Trust, Tonsberg, Norway; 4 Research Unit of Gynecology and Obstetrics, Department of Clinical Research, University of Southern Denmark, Odense, Denmark

**Keywords:** analgesia, childbirth, midwife, obstetrics, pudendal nerve block, suturing

## Abstract

**INTRODUCTION:**

Pudendal nerve block (PNB) is an effective analgesic during the second stage of labor and for suturing. With the introduction of epidural and spinal analgesia, PNB use decreased considerably. Most midwives receive some teaching on PNB during their midwifery education. The aim of this study was to examine the use of PNB by midwives in Norway.

**METHODS:**

This was a cross-sectional study, in January 2020, using an electronic questionnaire which was distributed to approximately 1500 midwives.

**RESULTS:**

A total of 527 midwives responded to the questionnaire (35%). Less than half (44.6%) of the midwives used PNB, of whom only half (123/235) used it frequently (at least once a month). The use of PNB was most common at specialized obstetric units with ≥1500 births per year. Midwives who reported good theoretical knowledge and practical skills of PNB used it significantly more often than midwives not reporting these (p<0.001). Reasons for not using PNB were: the lack of practice and experience (72.6%), and never having been taught (42.8%). Midwives reported needing training (83%) and clinical support to start using PNB (43%).

**CONCLUSIONS:**

Few midwives use PNB regularly. To increase the use of PNB, midwifery education needs to include both theoretical and practical skills teaching. Midwives with insufficient knowledge and skills require the same teaching and training. In the clinical area, midwives require clinical support and supervision to practice and gain experience. Women are not offered PNB as long as midwives are not confident in providing this method of pain relief.

## INTRODUCTION

Pudendal nerve block (PNB) is achieved by injecting local analgesia around the pudendal nerve^1,2^. The most common approach in obstetrics is transvaginal^1,3^. PNB provides good pain relief in the posterior part of the perineum and the inferior part of the vagina, and rectum^1,2^. Traditionally, PNB was recommended for assisted vaginal deliveries and suturing perineal injury in the absence of other adequate analgesia^1,4^. PNB has a rapid effect and there are few serious complications^1,2^. Despite knowledge of this effective method of pain relief, the use of PNB in childbirth has fallen over the last 40–50 years. Older research reports that 70% of all women who gave birth in Sweden in 1981 received a PNB^5^ while a survey in Germany from 1997 reported 23% use of PNB for vaginal birth in the Westfalen region^6^. A Norwegian prospective observational cohort study published in 2009 reported a prevalence of 10% of PNB in the common delivery ward, compared to 4% in the midwife led unit, among healthy primiparous women^7^. The use of PNB decreased as epidural and spinal analgesia became readily available^3^. The evidence is contradictory on the value or necessity of PNB in addition to epidural or spinal analgesia^8-10^. One older randomized study suggested that spinal analgesia was superior to PNB for operative vaginal delivery^9^. In contrast, a recent randomized study suggests that ultrasound-guided bilateral PNB may serve as an additional, effective adjunct method of labor analagia^8^. The Cochrane review on analgesia for forceps delivery concluded that there is insufficient evidence to support any particular analgesic agent or method as the most effective in providing pain relief for forceps delivery^10^.

The decline in the use of PNB has probably led to reduced practical skills in this method among clinicians^1,3^. The effect of PNB is contingent on the knowledge and skill of the provider^3,11^. An audit of 57 obstetricians evaluated their clinical technique against standards using both a questionnaire and adapted model pelvis^11^. The majority of participants were unable to describe correctly the point of infiltration and were unaware of the lag time required to reach adequate analgesia^11^. Both the Norwegian guidelines for care during labor and the NICE guidelines recommend PNB for operative vaginal births when the planned instrumental delivery is a low cavity forceps or vacuum extraction^12,13^. In the case of a middle or high cavity forceps or vacuum extraction, additional pain relief is recommended^13^. In the case of an instrumental delivery, it is likely a doctor who provides the PNB. However, these same Norwegian guidelines state that PNB offers an effective method of pain relief in the second stage of labor, after the membranes have gone and for suturing^13^. Thus, these guidelines do not exclude the use of PNB in spontaneous vaginal births. In the Nordic countries, providing PNB analgesia to relief pain in the second stage and for suturing has been part of the scope of midwifery practice^5,7,14^. To ensure that women can be offered this method of pain relief, training in the performance of PNB has to be part of the formal education and regular simulation training ought to be prioritized in the clinical setting to help improve and maintain practical skills^11,14^.

Currently, only two of the six midwifery education programs in Norway provide practical skill teaching and training in performing PNB, while all Norwegian midwifery education programs include PNB in the theoretical teaching on methods of pain relief. Together with the scarcity of new clinical studies on PNB use in childbirth, as well as no generally available records of PNB use in the Medical Birth Registry of Norway’s statistical bank^15^, this suggests that the use of PNB in practice may be limited in Norway. Against this background, the aim of this study was to document the use of PNB by midwives in Norway as well as their knowledge and skills in relation to this method of pain relief.

## METHODS

This study was cross-sectional using an anonymized electronic questionnaire available from https://nettskjema.no/. This semi-structured questionnaire consisted of 23 mandatory questions (Supplementary file). The questions were developed for this study and based on clinical expertise and available scientific literature. The first part of the questionnaire concerned background information including education, place of work, and demographics. The participants were in addition asked some general questions about PNB before the following question: ‘Do you use PNB in your work as a midwife?’. Those who answered ‘yes’ were asked seven questions about their use of PNB, while those who answered ‘no’ were asked two questions about why they did not use it.

The questionnaire was piloted in 20 persons, either midwives or student midwives. Feedback from the pilot test led to minor adjustments in the wording of the questions and answer options, before the questionnaire was distributed. The inclusion criteria for the study were midwives who worked in institutional maternity care in Norway and cared for women during birth. All maternity units in Norway were first contacted via telephone to ensure that the questionnaires could and would be sent out via work email. All maternity units agreed to forward the link to the questionnaire to relevant midwives at their unit. Subsequently, the consultant midwife at each maternity unit was contacted. Where a consultant midwife was not available, another contact midwife was asked to help with the distribution of the questionnaires.

Data were collected from 20 January to 13 February 2020. Based on information from the consultant/contact midwives, the questionnaires were forwarded to approximately 1500 midwives. To increase the response rate by reaching the midwives outside working hours, a link to the questionnaire was also shared on a closed midwifery Facebook group during the last week. The requirement to work in institutional practice with births was essential.

Prior to analysis, the collected data were merged into fewer categories. Anyone working less than 100% was categorized as ‘part-time’. The country of midwifery education was categorized as ‘Norway’ or ‘other’. The midwives were categorized according to the four regional health authorities in Norway as well as the type of maternity units in which they were working, which could have been a specialized obstetric unit, obstetric unit, maternity home, midwife-led unit, or other. Furthermore, the midwives were categorized according to the number of annual births at their workplace, and whether they held a Master’s degree or not (Table 1). The use of PNB was divided into three categories: ‘often’ which included weekly or monthly, ‘rarely’ for those who had used PNB less than once a month, and ‘never’. Training in PNB was covered during education to become a midwife, an in-house course, a private course, or from a colleague. For comparative analyses, it was coded as ‘had training’, while ‘no training’ and ‘don't remember’ was coded as ‘has not had training’. Similarly, for the question about clinical guideline for PNB in the workplace, ‘don't know’ was coded as ‘no guideline’. For questions about the extent of the midwife’s acquired theoretical knowledge and practical skills in relation to PNB, ‘very extensive’ and ‘extensive’ were merged into ‘extensive’. Similarly, ‘limited’ and ‘very limited/unskilled’ were merged into ‘limited’. The ‘in between’ category was denoted as ‘regular’ (Table 2).

**Table 1:** Characteristics of participating midwives (N=527)

*Characteristics*	*n (%)*
**Age** (years)	
≤30	55 (10.4)
31–40	165 (31.3)
41–50	154 (29.2)
≥51	153 (29.1)
**Experience as a midwife** (years)	
≤5	149 (28.3)
6–10	115 (21.8)
11–20	140 (26.6)
≥21	123 (23.3)
**Employment type**	
Full-time	257 (48.8)
Part-time	270 (51.2)
**Country of midwifery education**	
Norway	486 (92.2)
Other	41 (7.8)
**Education level**	
Master’s degree	131 (24.9)
**Regional health authority**	
South-Eastern Norway	287 (54.5)
Western Norway	94 (17.8)
Mid-Norway	71 (13.5)
Northern Norway	75 (14.2)
**Maternity unit**	
Specialized obstetric unit	278 (52.8)
Obstetric unit	213 (40.4)
Maternity home	18 (3.4)
Midwife-led unit	16 (3.0)
Other	2 (0.4)
**Births per year**	
1–499	103 (19.6)
500–1499	134 (25.4)
1500–2999	148 (28.1)
≥3000	142 (26.9)

**Table 2:** Midwives’ experience of pudendal nerve block (PNB) (N=527)

*Experience*	*n (%)*
**Use of PNB**	
Often	123 (23.3)
Rarely	112 (21.3)
Never	292 (55.4)
Received training*	
During midwifery education	233 (44.2)
In-house course	113 (21.4)
Private course	7 (1.3)
From a colleague	182 (34.5)
None	124 (23.5)
**Clinical guideline at workplace**	
Yes	327 (62.0)
No	200 (38.0)
**Theoretical knowledge of PNB**	
Extensive	247 (46.9)
Regular	90 (17.0)
Limited	190 (36.1)
**Practical skills of PNB**	
Extensive	184 (34.9)
Regular	54 (10.2)
Limited	289 (54.9)

* More than one answer option possible, thus some results are >100%.

### Statistical analysis

The distribution of demographic, educational and work-related characteristics was described using frequencies and percentages. Percentages and chi-squared tests were used to examine how midwives’ use, theoretical knowledge and practical experience with PNB were associated with age, seniority, employment type, place of education, education, regional health authority, maternity unit, number of births per year, training, clinical guideline for PNB, theoretical knowledge, and practical experience. Where cell numbers were five or less, Fisher’s exact test was used. All analyses were unadjusted except for the association between Master’s degree and use of PNB where we controlled for age and regional health authority in a supplementary analysis. A p<0.05 was considered to be statistically significant. The Statistical Package for the Social Sciences (SPSS) for Windows, version 26.0, was used for data analysis.

## RESULTS

A total of 527 midwives responded to the questionnaire corresponding to an estimated participation rate of 35%. The participants had an average age of >44 years and 13 years of experience (Table 1). One quarter of the participants had a Master’s degree. Over half of the participants worked for the South-Eastern Norway Regional Health Authority, nine in ten worked in an obstetric unit or a specialized obstetric unit, and most of the midwives worked in a unit with ≥1500 births per year.

More than half of the midwives had never performed a PNB (Table 2). Of those who had performed a PNB, 52% (123/235) performed a PNB at least once a month, while 48% (112/235) had performed this procedure less than once a month in the previous 6 to 12 months. Three-quarters of the midwives had received training in PNB. Over 60% stated that a clinical guideline for PNB was in place at their workplace. Almost half of the midwives reported extensive theoretical knowledge of PNB while one-third stated that they had extensive practical skills.

There was a strong association between midwives’ education and use of PNB as >35% of midwives with a Master’s degree used PNB often, compared to <20% of midwives without a Master’s degree (p=0.001) (Table 3). This association was attenuated after adjustment for age and regional health authority, but remained statistically significant. One in three midwives who worked in the South-Eastern Norway Regional Health Authority, at a specialized obstetric unit or on a maternity unit with 3000 or more births per year, used PNB often. In contrast, only one in ten midwives who worked in the Northern Norway Regional Health Authority, in a maternity home or a maternity unit with fewer than 500 births per year, used PNB often (p<0.001). Of those who had not received training in PNB or had no clinical guideline for use in the workplace, <10% used PNB, while one-third of those who had received training or had a clinical guideline at their workplace used PNB often (p<0.001). Almost all midwives who reported that they had limited theoretical knowledge and limited practical skills of PNB never used PNB. In contrast, PNB was often used by about half of the midwives who claimed to have extensive theoretical knowledge and practical skills of PNB (45% and 61%, respectively, p<0.001).

**Table 3:** Participant characteristics and use of pudendal nerve block (N=527)

*Characteristics*	*Often n (%) 123 (23.3)*	*Rarely n (%) 112 (21.3)*	*Never n (%) 292 (55.4)*	*p*
**Age** (years)				0.318
≤30	14 (11.4)	9 (8.0)	32 (11.0)	
31–40	46 (37.4)	32 (28.6)	87 (29.8)	
41–50	35 (28.5)	30 (26.8)	89 (30.5)	
≥51	28 (22.7)	41 (36.6)	84 (28.7)	
**Experience as a midwife** (years)				0.235
≤5	40 (32.5)	25 (22.3)	84 (28.8)	
6–10	25 (20.3)	22 (19.6)	68 (23.3)	
11–20	37 (30.1)	32 (28.6)	71 (24.3)	
≥21	21 (17.1)	33 (29.5)	69 (23.6)	
**Employment type**				0.412
Full-time	65 (52.8)	57 (50.9)	135 (46.2)	
Part-time	58 (47.2)	55 (49.1)	157 (53.8)	
**Country of midwifery education**				0.674
Norway	112 (91.1)	102 (91.1)	272 (93.2)	
Other	11 (8.9)	10 (8.9)	20 (6.8)	
**Education level**				0.001
Master’s degree	46 (37.4)	24 (21.4)	61 (20.9)	
No Master’s degree	77 (62.6)	88 (78.6)	231 (79.1)	
**Regional health authority**				<0.001
South-Eastern Norway	98 (79.4)	51 (45.6)	138 (47.3)	
Western Norway	10 (8.1)	30 (26.8)	54 (18.5)	
Mid-Norway	9 (7.3)	23 (20.5)	39 (13.4)	
Northern Norway	6 (4.9)	8 (7.1)	61 (20.8)	
**Maternity unit**				<0.001
Specialized obstetric unit	2 (1.6)	1 (0.8)	15 (5.1)	
Obstetric unit	24 (19.5)	46 (41.1)	143 (49.0)	
Maternity home	94 (76.5)	61 (545)	123 (42.1)	
Midwife-led unit	2 (2.4)	4 (3.6)	9 (3.1)	
Other	0 (0.0)	0 (0.0)	2 (0.7)	
**Births per year**				<0.001
1–499	1 (0.8)	17 (15.2)	85 (29.1)	
500–1499	17 (13.8)	28 (25.0)	89 (30.5)	
1500–2999	59 (48.0)	29 (25.9)	60 (20.5)	
≥3000	46 (37.4)	38 (33.9)	58 (19.9)	
**Received training**				<0.001
Yes	123 (100)	111 (99.1)	169 (57.9)	
No	0 (0.0)	1 (0.9)	123 (42.1)	
**Clinical guideline at workplace**				<0.001
Yes	109 (88.6)	85 (75.9)	133 (45.5)	
No	14 (11.4)	27 (24.1)	159 (54.5)	
**Theoretical knowledge of PNB**				<0.001
Extensive	11 1 (90.3)	83 (74.1)	53 (18.2)	
Regular	10 (8.1)	24 (21.4)	56 (19.2)	
Limited	2 (1.6)	5 (4.5)	183 (62.7)	
**Practical skills of PNB**				<0.001
Extensive	112 (91.1)	64 (57.1)	8 (2.7)	
Regular	10 (8.1)	29 (25.9)	15 (5.1)	
Limited	1 (0.8)	19 (17.0)	269 (92.2)	

Among midwives with a Master’s degree, 59% reported extensive theoretical knowledge and 48% reported extensive practical skills (Table 4). For midwives without a Master’s degree, the corresponding numbers were 43% and 31% (p<0.001). While 54% of midwives in the South-Eastern Norway Regional Health Authority reported to have extensive theoretical knowledge of PNB and 44% to have extensive practical skills, this applied to just 24% and 15% of midwives in the Northern Norway Regional Health Authority (p<0.001). There were strong associations between having received training and a clinical guideline present at the workplace and the theoretical knowledge and practical skills of the midwives (p<0.001).

**Table 4:** Associations between midwives’ knowledge and experience with pudendal nerve block (N=527)

	*Theoretical knowledge of PNB*				*Practical skills of PNB*			
	*Extensive n (%) 247 (46.9)*	*Regular n (%) 90 (17.0)*	*Limited n (%) 190 (36.1)*	*p*	*Extensive n (%) 184 (34.9)*	*Regular n (%) 54 (10.2)*	*Limited n (%) 289 (54.9)*	*p*
**Age** (years)				0.863				0.418
≤30	24 (43.6)	9 (16.4)	22 (40.0)		17 (30.9)	3 (5.5)	35 (63.6)	
31–40	73 (44.2)	32 (19.4)	60 (36.4)		59 (35.8)	17 (10.3)	89 (53.9)	
41–50	73 (47.4)	23 (14.9)	58 (37.7)		47 (30.5)	20 (13.0)	87 (56.5)	
≥51	77 (50.3)	26 (17.0)	50 (32.7)		61 (39.9)	14 (9.1)	78 (51.0)	
**Experience as a midwife** (years)				0.264				0.135
≤5	65 (43.6)	26 (17.4)	58 (39.0)		51 (34.2)	9 (6.0)	89 (59.8)	
6–10	48 (41.7)	20 (17.4)	47 (40.9)		32 (27.8)	17 (14.8)	66 (54.4)	
11–20	70 (50.0)	29 (20.7)	41 (29.3)		53 (37.9)	17 (12.1)	70 (50.0)	
≥21	64 (52.0)	15 (12.2)	44 (35.8)		48 (39.0)	11 (8.9)	64 (52.1)	
**Employment type**				0.295				0.250
Full-time	127 (49.4)	46 (17.9)	84 (32.7)		93 (36.2)	31 (12.0)	133 (51.8)	
Part time	120 (44.4)	44 (16.3)	106 (39.3)		91 (33.7)	23 (8.5)	156 (57.8)	
**Country of midwifery education**				0.070				0.613
Norway	224 (46.1)	88 (18.1)	174 (35.8)		171 (35.2)	48 (9.9)	267 (54.9)	
Other	23 (56.1)	2 (4.9)	16 (39.0)		13 (31.7)	6 (14.6)	22 (53.7)	
Education level				0.005	0.003			0.003
Master’s degree	77 (58.8)	20 (15.2)	34 (26.0)		62 (47.3)	12 (9.3)	57 (43.5)	
No Master’s degree	170 (42.9)	70 (17.7)	156 (39.4)		122 (30.8)	42 (10.6)	232 (58.6)	
**Regional health authority**				<0.001				<0.001
South-Eastern Norway	154 (53.7)	46 (16.0)	87 (30.3)		126 (43.9)	26 (9.1)	135 (47.0)	
Western Norway	42 (44.7)	16 (17.0)	36 (38.3)		28 (29.8)	11 (11.7)	55 (58.5)	
Mid-Norway	33 (46.5)	18 (25.4)	20 (28.1)		19 (26.8)	12 (16.9)	40 (56.3)	
Northern Norway	18 (24.0)	10 (13.3)	47 (62.7)		11 (14.7)	5 (6.7)	59 (78.6)	
**Maternity unit**				<0.001				<0.001
Specialized obstetric unit	154 (55.4)	48 (17.3)	76 (27.3)		121 (43.5)	32 (11.5)	125 (45.0)	
Obstetric unit	82 (38.5)	38 (17.8)	93 (43.7)		54 (25.4)	19 (8.9)	140 (65.7)	
Maternity home	3 (16.7)	1 (5.6)	14 (77.7)		3 (16.7)	0 (0.0)	15 (83.3)	
Midwifery-led unit	8 (50.0)	2 (12.5)	6 (37.5)		6 (37.5)	3 (18.8)	7 (43.7)	
Other	0 (0.0)	1 (50.0)	1 (50.0)		0 (0.0)	0 (0.0)	2 (100.0)	
**Births per year**			<0.001					<0.001
1–499	27 (26.2)	14 (13.6)	62 (60.2)		12 (11.7)	6 (5.8)	85 (82.5)	
500–1499	52 (38.8)	30 (22.4)	52 (38.8)		34 (25.4)	18 (13.4)	82 (61.2)	
1500–2999	83 (56.1)	26 (17.6)	39 (26.3)		73 (49.3)	16 (10.8)	59 (39.9)	
≥3000	85 (59.9)	20 (14.1)	37 (26.0)		65 (45.7)	14 (9.9)	63 (44.4)	
**Received training**				<0.001				<0.001
Yes	235 (58.3)	73 (18.1)	95 (23.9)		183 (45.5)	52 (12.9)	168 (41.6)	
No	12 (9.7)	17 (13.7)	95 (76.6)		1 (0.8)	2 (1.6)	121 (97.6)	
**Clinical guideline at workplace**				<0.001				<0.001
Yes	208 (63.6)	57 (17.4)	62 (19.0)		152 (46.5)	43 (13.1)	132 (40.4)	
No	39 (19.5)	33 (16.5)	128 (64.0)		32 (16.0)	11 (5.5)	157 (78.5)	
**Theoretical knowledge of PNB**								<0.001
Extensive					167 (67.6)	28 (11.6)	52 (21.2)	
Regular					23 (25.5)	53 (58.9)		
Limited					3 (1.6)	3 (1.6)	184 (96.8)	
**Practical skills of PNB**				<0.001				
Extensive	167 (90.8)	14 (7.6)	3 (1.6)					
Regular	28 (51.8)	23 (42.6)	3 (5.6)					
Limited	52 (18.0)	53 (18.3)	184 (63.7)					

**Figure 1:**
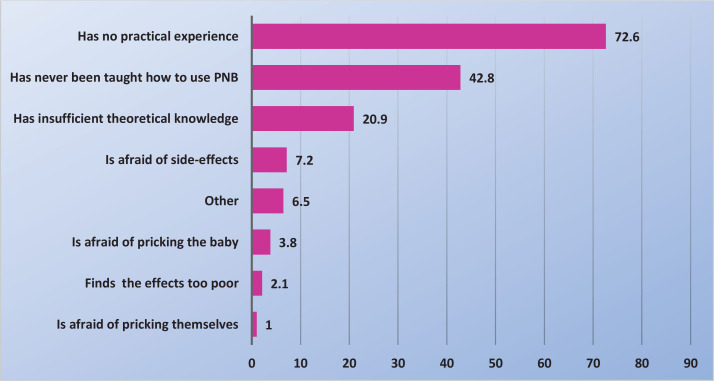
Reasons (%) why the midwives did not use PNB (N=292)

**Figure 2:**
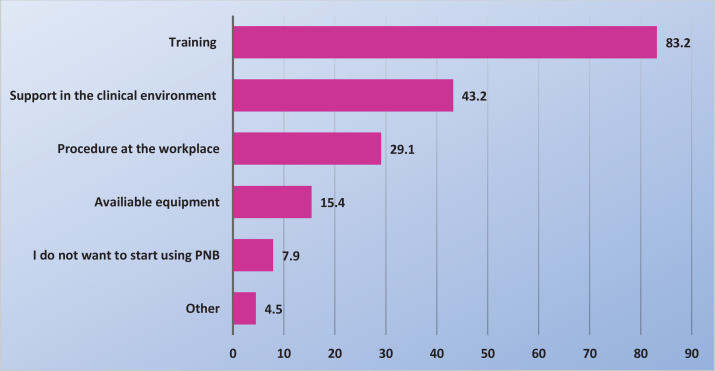
What was needed (%) for midwives to start using PNB (N=292)

More than 70% of midwives said that they did not use PNB because of lack of practical experience while nearly half of the midwives said that they never received training in the use of PNB (Figure 1). Figure 2 displays measures reported by the midwives that might help them start using PNB. Only 8% of midwives, who did not use PNB, also did not want to start using it. The most common indications for midwives to use PNB were to relief pain in the expulsion phase [67% (157/235)], an early urge to push [60% (142/235)] and if, for various reasons, epidural analgesia was not possible [57% (134/235)], while 49% (115/235) used PNB in connection with suturing (data not shown in tables or figures). Over 85% of midwives who used PNB were largely satisfied or very satisfied with the effect, regardless of whether they used it during the delivery or for suturing. By contrast, <2% of midwives who used PNB were largely dissatisfied or very dissatisfied with the effect. Fifty percent of the midwives reported having observed obstetricians providing pudendal nerve block analgesia.

## DISCUSSION

In this national cross-sectional study of midwives caring for women during birth in maternity care in Norway, less than 25% of midwives used PNB often. It was apparent that midwives, who had received training in PNB or had a clinical guideline for PNB in the workplace, used PNB to a significantly greater extent than other midwives. Strong associations were observed between midwives having gained theoretical knowledge and practical skills and their use of PNB.

The Medical Birth Registry of Norway’s statistical bank, accessible to the public, does not provide information on the prevalence of PNB use^15^. Upon request, we were informed that the prevalence of PNB use was 2.1% in 2012 and 6.4% in 2018. Although this shows a significant increase, it still seems surprising that almost half of the midwives in our study reported using PNB. This apparent discrepancy can in part be explained by a minority of midwives using PNB often. In addition, midwives using PNB often, may be more likely to respond to a questionnaire on this topic. Finally, at one of the largest hospitals in the South-Eastern Region of Norway, where most of the participating midwives worked, a Swedish midwife has re-introduced PNB through in-house training courses, clinical support and supervision^14^. At the same hospital, a PhD project is being conducted into the effect and side-effects of PNB. The first publication in this project shows that the use of PNB at this hospital (one hospital with two sites) is substantially higher than the national level^16^.

The national guidelines in Norway are in contrast to the NICE guidelines, which recommend PNB for operative births only and do not seem to allow for the use of PNB in spontaneous birth, administered by midwives^12,13^. Health professionals in Norway are used to consulting the NICE guidelines when writing their own. However, while there are similarities, there are also considerable cultural, organizational and historical differences between the UK and Norway in the way care of laboring women is provided. Such differences can influence guidelines. Furthermore, in addition to research, evidence-based practice takes into account the practitioner’s clinical experience, patient experience, and information from the local context^17^. Thus, the historical tradition of midwives providing PNB in Norway influences the clinical skills available and the scope of their practice.

How pain during labor is viewed, expressed and controlled is subject to cultural variations^18^. In the midwifery literature the control of pain during normal labor has been summarized in two contrasting paradigms^19^. There is the model which is based on the ideology of promoting normal birth which encourages midwives to think in terms of ‘working with pain’ rather than trying to take it away^19^. On the other hand, there is the ‘pain relief’ approach which has been described as a model in which midwives offer a variety of methods that can be used to take the pain away^19^. Laboring women may belong to either paradigm and midwives need to be able to care for both, women who choose to work with pain and those who choose to have pain relief. Thus, adding the skill of being able to administer a PNB increases midwives’ ability to meet more women’s needs. Women’s needs being ignored and the experience of pain beyond control are associated with a negative birth experience^20^. A negative birth experience may result in fear of childbirth and request for birth by cesarean section in a subsequent pregnancy^21^. While PNB has been described as a relatively simple procedure with few complications, it does involve locating the correct anatomical site, the use of a long needle, and the insertion of analgesia^3,22^. In addition, situations calling for a PNB during birth are often stressful such as inadequate pain relief or imminent operative delivery. Thus, it is not surprising, and may even be regarded good clinical judgement, that the midwives in our study, who reported they lacked adequate teaching and training, did not use PNB. Midwifery educational programs use simulation and skills training to prepare for clinical practice^23^. Research suggests that simulation and skills training creates a link between theory and practice^23,24^. Students value repetitive practices in a safe and secure environment allowing learning from mistakes without risk to the patient^24^. Simulation and skills training gives students confidence and facilitates clinical practice^24^. The review by Cooper et al.^23^ showed that simulation-based learning in midwifery is commonly used for obstetric emergencies but did not mention PNB. However, a publication from Portugal suggests the use of obstetric simulators to train for this procedure^25^.

In our study, midwives who worked at units with fewer than 500 births used PNB significantly less frequently than midwives at units with more births. This may be because they support fewer women during birth and thus require this method of pain relief less often. However, there is a risk for health professionals becoming insecure in performing skills that they do not use frequently. Simulation skills training is already established for keeping obstetricians and midwives’ skills up-to-date for obstetric emergencies and could be used for PNB^25,26^. Another possible reason why midwives in large and specialized units use PNB more than in smaller units could be that small units focus more on non-pharmacological pain relief throughout the birthing process, as the rates of epidural and spinal suggest^15^. However, women in such units could benefit from being offered a PNB for suturing^27,28^. Interventions are reported to lead to more interventions^29^. In specialized obstetric units, midwives are likely to be more familiar with performing other interventions and may thus use PNB more easily^30^.

Less than half of the midwives in our study reported having received teaching on the use of PNB during their midwifery education. While this may be disappointing, continued professional development (CPD) is a necessity for all nurses and midwives^31^. Our findings that midwives have learned how to use PNB from a colleague or in-house training is in agreement with the evidence from a recent umbrella review concluding that knowledge and skills acquired through CPD are often transferred into practice^32^. The midwives in our study that had not been using PNB indicated that learning clinical skills and training was necessary for them to start using PNB.

It was apparent from the study that PNB was used significantly more often in units that had a clinical guideline for the use of PNB in place, compared to units that did not. Other research shows that the presence of evidence-based guidelines can strengthen midwifery practice^33^. The national guidelines for obstetric care mention PNB in one paragraph only in their chapter on pain relief in labour^13^. Thus, a local clinical guideline for PNB signals a positive view of the use of PNB and is likely authored by local clinicians with expertise in this area. A local clinical guideline not only provides relevant information but suggests clinical support for this procedure. Midwives in our study mentioned the need for clinical support in order to start using PNB. A study from Oslo shows that a local advocate organizing in-house training and clinical support increases the use of PNB^14,16^. Collaboration between midwives and doctors in clinical support for this procedure would enhance the opportunities for midwives to practice this skill.

### Strengths and limitations

This was a national study that invited the participation of the entire population of midwives in Norwegian institutional maternity units caring for women during birth. The proportion of the participants from each Regional Health Authority corresponds well with the number of births in these regions (Supplementary file, Table 1). The midwives participating in this study were slightly younger than those responding to another recent national survey^34^. However, that survey did not restrict its participants to midwives caring for women during labor. It seems that older midwives move away from care of women in active labor to positions without shift and weekend duties^34^. Thus, a study on PNB would most likely recruit younger midwives. A limitation is that the study was only available for approximately 3 weeks. Thus, the estimated participation rate of 35% is rather low. However, over 500 midwives participated from a total workforce of approximately 3000 midwives, of whom a substantial number work in areas where providing PNB in labor is not relevant^34^. This suggests that our findings are generalizable to midwives caring for women in labor. By sharing the link on social media, some participants not meeting the inclusion criteria for the study may have responded to it despite clear instructions. This could have increased the number of participating midwives not using PNB. On the other hand, there may be a risk of selection bias if participating midwives had a particular interest in PNB, increasing the number of those using PNB. All information about midwives’ skills, knowledge and experience with PNB was self-reported and may have led to both over- and under-reporting. This is a potential normal consequence of self-reporting, and it is important to take that into consideration. At the same time, midwives’ self-perception of their skills is an important objective for understanding their use of PNB. This is the first study to document midwives’ use, knowledge and skills of PNB in Norway, consequently there are no studies available for comparison.

## CONCLUSIONS

This study found that less than half of midwives caring for women during birth in Norway used PNB. One in four midwives used it often, at least once a month, and the majority of these midwives reported good theoretical knowledge and practical skills of PNB. Emerging literature suggests that there is a renewed and increasing interest in the use of PNB in birth^1,8,14,18^, and our study shows that the majority of midwives, not currently using it, would like to start using PNB. Theoretical education and clinical skills training as well as support from the clinical environment and a clinical guideline at the workplace are considered to be important factors in midwives’ knowledge and use of PNB. Further research is needed to explore women’s experience with receiving a PNB for both spontaneous and instrumental birth.

## Data Availability

The data supporting this research are available from the authors on reasonable request.
